# Genome and Transcriptome Analyses Provide Insight Into the Omega-3 Long-Chain Polyunsaturated Fatty Acids Biosynthesis of *Schizochytrium limacinum* SR21

**DOI:** 10.3389/fmicb.2020.00687

**Published:** 2020-04-16

**Authors:** Limin Liang, Xuehai Zheng, Wenfang Fan, Duo Chen, Zhen Huang, Jiangtao Peng, Jinmao Zhu, Weiqi Tang, Youqiang Chen, Ting Xue

**Affiliations:** ^1^The Public Service Platform for Industrialization Development Technology of Marine Biological Medicine and Products of the State Oceanic Administration, Center of Engineering Technology Research for Microalga Germplasm Improvement of Fujian, Fujian Key Laboratory of Special Marine Bioresource Sustainable Utilization, Key Laboratory of Developmental and Neural Biology, Southern Institute of Oceanography, College of Life Sciences, Fujian Normal University, Fuzhou, China; ^2^Institute of Oceanography, Marine Biotechnology Center, Minjiang University, Fuzhou, China

**Keywords:** *Schizochytrium limacinum* SR21, genome, transcriptome, fatty acid, DHA

## Abstract

*Schizochytrium* sp. is the best natural resource for omega-3 long-chain polyunsaturated fatty acids. We report a high-quality genome sequence of *Schizochytrium limacinum* SR21, which has a 63 Mb genome size, with a contig N50 of 2.67 Mb and 6,838 protein-coding genes. Phylogenomic and comparative genomic analyses revealed that DHA-producing *Schizochytrium* and *Aurantiochytrium* strains were highly similar and possessed similar genes. Analysis of the fatty acid synthase (FAS) for LC-PUFAs production results in the annotation of all genes in map00062 and map01212. A gene cluster and 10 ORFs related to PKS pathway were found in the genome. 1,402 differentially expressed genes (DEGs) of the treated groups (0.5 g/L yeast extract) were identified by comparing with the control groups (1.0 g/L yeast extract) at 36 h. A weighted gene coexpression network analysis revealed that 2 of 7 modules correlated highly with the fatty acid and DHA contents. The DEGs and transcription factors were significantly correlated with fatty acid biosynthesis, including MYB, Zinc Finger and ACOX. The results showed that these hub genes are regulated by genes involved in fatty acid biosynthesis pathways. The results providing an important reference for further research on promoting fatty acid and DHA accumulation in *S. limacinum* SR21.

## Introduction

*Schizochytrium* sp. is a unicellular fungal-like marine protist found ubiquitously in marine environments and considered as the best natural resource for omega-3 long-chain polyunsaturated fatty acids (LC-PUFAs) (Newell et al., 2019). Docosahexaenoic acid (DHA, 22:6) is a high-value LC-PUFA with strong biological activity for the food, feed, medicine, health care, and pharmaceutical industries (Ambati et al., 2014; Browning et al., 2014; Newell et al., 2019). The traditional commercial source of DHA is fish oil, which is undesirable because of its offensive odor, potential contamination and complex purification process (Mühlroth et al., 2013; Ye et al., 2015). *Schizochytrium* sp. is considered a noteworthy and satisfactory alternative to fish oil due to the advantages of its fast growth rate, high purity, slight fishy smell, and more than 50% lipid rich in DHA. Many studies have been conducted to optimize the media composition and culture conditions, including nutrient deprivation, strain adaption, temperature shift and genetic engineering to enhance the lipid accumulation efficiency in *Schizochytrium* sp. (Sakaguchi et al., 2012; Ren et al., 2014, 2015; Sun et al., 2014, 2016). However, *Schizochytrium* sp. can accumulate lipids up to 50% of its dry weight, with DHA generally constituting 40% or more of the total oils by the above mentioned methods.

Two LC-PUFAs biosynthetic pathways, fatty acid synthase (FAS) and polyketide synthase (PKS) system, have long been speculated to exist in the genomes of *Aurantiochytrium* sp., *Schizochytrium* sp., and *Thraustochytriidae* sp. (Warude et al., 2006; Orikasa and Nishida, 2007). The two major steps in fatty acid biosynthesis are elongation and desaturation carried out by two carbon units in the FAS pathway (Morais et al., 2015). For FAS pathway, Lippmeier et al., 2009 found Δ-5, Δ-6, and Δ-9 elongase activities in *Schizochytrium* sp. ATCC20888 without the present of Δ-12 desaturation. Ren et al. (2017), detected one elongase and three kinds (Δ-6, Δ-8, and Δ-12) of desaturase activities in *Schizochytrium* sp. PKS pathway of LC-PUFAs synthesis involve the processing of the saturated 16:0 or 18:0 products by repetitive decarboxylative Claisen ester condensations. This process usually involves 3-ketoacyl synthase (KS), malonyl-CoA acyltransferase (MAT), acyl carrier proteins (ACP), 3-ketoacyl-ACP reductase (KR), enoyl reductase (ER), and dehydrase (DH). Metz (2001), identified 11 regions within the five open reading frames (ORFs) form *Shewanella* sp., eight of these were related to PKS proteins and three of these to FAS. Meanwhile, three ORFs (orfA, orfB, orfC) involved in the synthesis of LC-PUFAs genes from *Schizochytrium* sp. were identified and had similar structural and functional regions of genes compared with *Shewanella* sp. by sequencing analysis and comparison (Metz, 2001). FAS and PKS biosynthetic pathways have strong homologies in the chemical mechanisms involved in chain elongation and precursors (acetyl-CoA, malonyl-CoA) (Kaulmann and Hertweck, 2002). Although clustered genes involved FAS and PKS pathway were analyzed based on the genomic fragment, transcriptomics and incomplete genomic information, the complete FAS and PKS genes of DHA-producing *Schizochytrium* sp. have not been reported yet by high-quality genomic resources.

In order to uncover the regulatory mechanism of lipid migration and LC-PUFA synthesis, genomics and transcriptomics studies have revealed genes or proteins involved in LC-PUFA biosynthesis (Ren et al., 2017). Scaffolding genome assemblies remain challenging even with the rapidly increasing sequence coverage generated by current next-generation sequence technologies. With scaffolding information, draft genome sequences of four DHA-producing thraustochytrid strains, namely, *Schizochytrium* sp. CCTCC M209059 (39.09 Mb, scaffold N50 of 595 kb), *Aurantiochytrium* sp. T66 (43 Mb, scaffold N50 of 1.3 Mb), *Schizochytrium* sp. Mn4 (65.69 Mb, scaffold N50 of 153 kb) and *Thraustochytriidae* sp. SW8 (61.67 Mb, scaffold N50 of 127 kb), have been produced and provided some key information to improve our understanding of the molecular mechanisms for LC-PUFA synthesis (Ji et al., 2015; Liu et al., 2016; Song et al., 2018), but incomplete genome assemblies bring some problems for the subsequent study of *Schizochytrium* sp. LC-PUFA biosynthesis at the DNA level. Meanwhile, high-quality genomic resources will help to breed novel strains of *Schizochytrium* sp. that could have higher LC-PUFAs and DHA yield in industries. In addition, a systematic analysis of the molecular synthesis and regulatory networks for PUFA biosynthesis in *Schizochytrium* sp. has not been performed by genomics and transcriptomics.

In this study, to systematically understand the molecular pathway and regulatory network for LC-PUFAs and DHA production in *Schizochytrium* sp., we used third-generation sequencing (TGS) of the PacBio SEQUEL platform to generate a high-quality genome assembly and annotation of the DHA-producing strain *Schizochytrium limacinum* SR21 (*S. limacinum* SR21). In addition, LC-PUFA production could be enhanced under yeast extract starvation, which is the most common stress that occurs during *Schizochytrium* sp. fermentation. We therefore performed gene family, transcriptome sequencing and weighted correlation network analysis (WGCNA) on stressed cells at six stages of fermentation (12, 24, 36, 48, 60, and 72 h) to reveal additional genes that are potentially involved in the accumulation and regulation of LC-PUFAs and DHA production.

## Materials and Methods

### Sample Materials, Genomic DNA Extraction, and Genome Assembly

*Schizochytrium limacinum* SR21 (*S. limacinum* SR21) was purchased from the American Type Culture Collection (ATCC). The basal fermentation medium contained 5.0 g of glucose, 1.0 g of peptone, 1.0 g of yeast extract, and 1 L of seawater. Cells were grown in 250-mL Erlenmeyer flasks containing 100 mL of medium and incubated at 20°C in an orbital shaker set at 220 rpm. Genomic DNA was isolated from 100 ml of fresh culture. The cell suspension was centrifuged at 8000 rpm for 5 min. The cell pellet was then suspended in approximately 1 mL of medium and pipetted into a 2-mL tube and centrifuged again at 8000 rpm for 5 min. Briefly, 800 μL of 2% mercaptoethanol solution was pipetted into each sample, followed by the addition of 800 μL of 10% w/v CTAB (cetyl/hexadecyl trimethyl ammonium bromide, in 0.7M NaCl solution) and incubation at 56°C for 10 min. After extraction with an equal volume of phenol: chloroform: isoamyl alcohol (25:24:1), the mixture was centrifuged at 12,000 rpm for 10 min twice. The supernatant was dissolved in 0.4 mL of 100 μg/mL RNase and incubated at 37°C for 30 min. An equal volume of chloroform: isoamyl alcohol (24:1) was then centrifuged at 12,000 rpm for 10 min. Genomic DNA was precipitated by adding 2.5 volumes of 100% ethanol and collected by spinning at 12,000 rpm and 4°C for 10 min. After the supernatant was discarded, the resulting genomic DNA pellet was stored in 5.0 mL of 70% cold ethanol at 4°C overnight to allow the impurity to dissolve. Finally, DNA was eluted in 100 μL of 10 mM Tris-HCl by centrifugation at 12,000 rpm for 1 min. The purity and concentration of DNA were analyzed using a NanoDrop 2000 Spectrophotometer (Thermo Scientific, United States).

More than 5 μg of sheared and concentrated DNA was applied to size-selection by the BluePippin system. Approximately 20-kb SMRTbellTM libraries were prepared according to the released protocol from PacBio company. A total of 6.7 Gb subreads were sequenced on the PacBio sequel system, i.e., 106 × coverage of the estimated genome size. After removing the low-quality (containing 10 or more Ns and low-quality bases with quality scores ≤ 7) and redundant reads, the full PacBio subreads were corrected, trimmed and assembled using CANU version 1.7 with parameter corOutCoverage = 80 (Koren et al., 2017). To improve the accuracy, primary contigs were further polished by the Pilon program using 9.4 Gb (150×) Illumina paired-end reads (Walker et al., 2014; Supplementary Table S1). Assessment of genome completeness was performed with BUSCO using Eukaryotic models (Simao et al., 2015).

### Genome Annotation

Before annotating the gene structures of the *S. limacinum* SR21 genome, we identified repeat sequences using multiple programs, including Tandem Repeats Finder, LTR_FINDER, Repeat ProteinMask and RepeatMasker (Huda and Jordan, 2009). Tandem Repeats Finder was employed to search for tandem repeats in our genome assembly using the following parameters: Match = 2, Mismatch = 5, Delta = 7, PM = 60, PI = 10, Minscore = 80, and MaxPerid = 2,000. A *de novo* repeat library was built by the LTR_FINDER (version 1.0.6). Subsequently, the RepeatMasker was utilized to align our genome sequences onto the Repbase TE (version 3.2.9) to search the known repeat sequences as well as map onto the *de novo* repeat libraries to identify novel types of repeat sequences (Jurka et al., 2005).

We then performed annotation of the *S. limacinum* SR21 genome assembly with three approaches, including homology-based, transcriptome-based, and *ab initio* annotation. We selected several representative species, including *Paramecium tetraurelia*, *Saccharomyces cerevisiae*, *Symbiodinium kawagutii* and *Symbiodinium minutum*, *Chlamydomonas eustigma*, *Chromochloris zofingiensis*, and *Micromonas pusilla*, to perform the homology annotation (Brussaard et al., 1999; Kellis et al., 2004; Aury et al., 2006; Lin et al., 2015; Kanzaki et al., 2017; Roth et al., 2017). The protein sequences from the abovementioned species were aligned onto our genome sequences utilizing TblastN with an E-value ≤ 1e-5. Genewise 2.2.0 was subsequently employed to predict possible gene structures based on all TblastN results (Smith et al., 2011; Kagale et al., 2014). Total RNA was extracted from control cells for subsequent transcriptome sequencing using an Illumina HiSeq 4000 platform. We utilized Cufflinks (version 2.2.1) to identify the preliminary genes (Trapnell et al., 2012). Moreover, Augustus and Genscan were selected for *ab initio* annotation using the repeat-masked genome sequences (Thayer et al., 2000; Sommerfeld et al., 2009). Finally, we employed GLEAN software to integrate all genes predicted from the three annotation procedures (Elsik et al., 2007). Functional annotation of the protein-coding genes was carried out by BLASTP with an E-value ≤ 1e-5 to four integrated protein sequence databases: eggNOG, GO, COG, and KEGG (Ashburner et al., 2000; Tatusov et al., 2000; Kanehisa et al., 2014).

### Phylogenetic Analysis

To investigate the relationship of *S. limacinum* SR21 with the other 13 species, we performed phylogenetic analysis using the protein-coding gene from the *S. limacinum* SR21 genome and other species. Protein sequences of single-copy genes were extracted from 14 species and downloaded from the NCBI and JGI databases, including *Arabidopsis thaliana* (*A. thaliana*), *Aurantiochytrium limacinum* ATCC-MYA-1381 (*A. limacinum* ATCC-MYA-1381), *Chlamydomonas reinhardtii* (*C. reinhardtii*), *Fragilariopsis cylindrus* (*F. cylindrus*), *Hondaea fermentalgiana* (*H. fermentalgiana*), *Phaeodactylum tricornutum* (*P. tricornutum*), *Phytophthora infestans* (*P. infestans*), *Phytophthora parasitica* (*P. parasitica*), *Phytophthora sojae* (*P. sojae*), *Saprolegnia parasitica* (*S. parasitica*), *Schizochytrium aggregatum*-ATCC28209 (*S. aggregatum*-ATCC28209), *Thalassiosira pseudonana* (*T. pseudonana*) and *Thraustotheca clavata* (*T. clavata*). The similarities among proteins from all species were searched using the all-to-all manner by BLASTP software with an E-value ≤ 1e-5. Orthofinder software (version 2.27) was used to generate multiple sequence alignment for protein sequences in each single-copy family with default parameters as well as phylogenetic tree construction (Emms and Kelly, 2015). *A. thaliana* and *C. reinhardtii* were designated as the outgroup of the phylogenetic tree. The phylogenetic relationships were constructed through superalignment of the coding DNA sequences (CDSs) using the maximum likelihood (ML) method. The CDSs were aligned with the guidance of the protein alignments and then concatenated into the superalignment matrix of each family. We compared the cluster size differences between the ancestor and each species, analyzed the expansion and contraction of the gene families by using CAFE software (version 2.1) (Cristianini and Demuth, 2006).

### Culture Conditions and Induction of DHA Biosynthesis

**FIGURE 1:**
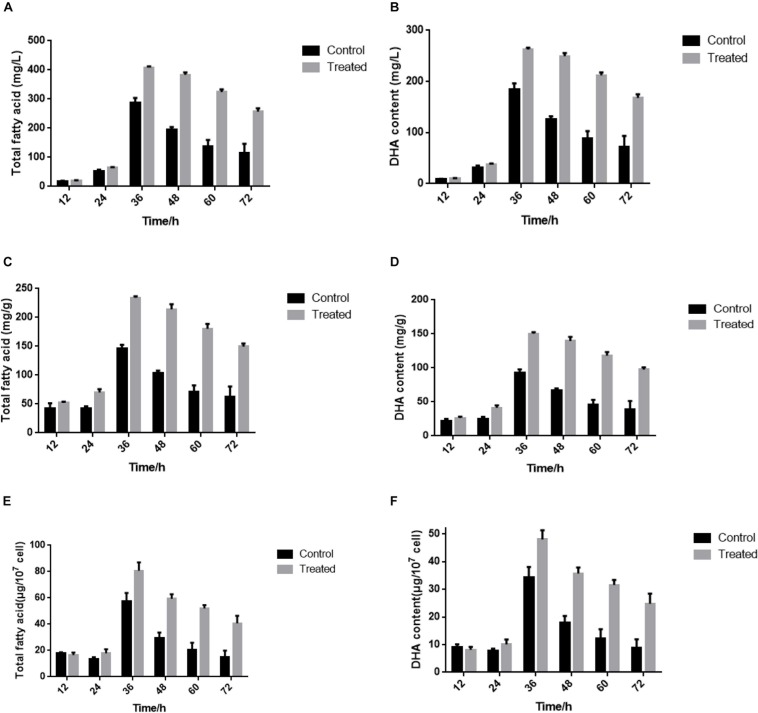
**(A,B)** Content of total fatty acid and DHA in fresh weight at different cultivation times. **(C,D)** Content of total fatty acid and DHA in dry weight at different cultivation times. **(E,F)** Content of total fatty acid and DHA in 10^7^ cell density at different cultivation times. The control groups represent that *S. limacinum* SR21 was cultured in basal fermentation medium with 1.0 g/L yeast extract. The treated groups represent that *S. limacinum* SR21 was cultured in basal fermentation medium with 0.5 g/L yeast extract. All experiments were performed in triplicate. Each value presents the mean ± SD. “*I*” represents error bars for the various determinations (*n* = 3).

Recent studies showed that yeast extract starvation could enhance lipid production, which is essential for DHA accumulation (Konishi et al., 2011). In our previous study, the total fatty content and DHA production of *S. limacinum* SR21 drastically increased from 12 to 36 h in basal fermentation medium containing 1.0 or 0.5 g/L yeast extract, respectively. However, the stimulation of total fatty content and DHA production was gradually attenuated above 36 h (Figure 1). Compared with 1.0 g/L yeast extract, 0.5 g/L yeast extract could be conducive to the accumulation of DHA. Therefore, we considered that the attenuation of yeast extract-induced stimulation may be caused by the shortage of carbon sources during cultivation with 0.5 g/L yeast extract. To investigate the influence of yeast extract on lipid production, *S. limacinum* SR21 was cultured in basal fermentation medium with 0.5 g/L yeast extract. *S. limacinum* SR21 was cultured in 500-mL beakers in basal fermentation medium under a light intensity of 25 mmol photons m^–1^ s^–2^ with a 12 h:12 h light:dark cycle at 20°C. When the cells reached the logarithmic phase (10^6^ cells/mL), the culture was evenly divided into six aliquots of 1000 mL each at different concentrations of yeast extract. They were used as three experimental controls (control, 1.0 g/L yeast extract) and three treatments (treated, 0.5 g/L yeast extract) and were sampled at 12, 24, 36, 48, 60, and 72 h. All the samples were centrifuged at 8,000 rpm, kept at 4°C for 10 min, and then stored at −80°C until subsequent RNA-seq analyses.

### Total RNA Isolation, Library Construction, and Sequencing

Analysis of the influence of yeast extract on lipid production of *S. limacinum* SR21 was performed in basal fermentation medium at different concentrations of yeast extract. Samples from the six stages were collected at 12, 24, 36, 48, 60, and 72 h. The collected samples were immediately frozen in liquid nitrogen and stored at −80°C until RNA extraction. The samples of stages 12, 24, 36, 48, 60, and 72 h of *S. limacinum* SR21 were used to construct six libraries. Total RNA from *S. limacinum* SR21 was extracted using a TransZol Up Plus RNA Kit (Transgen Biotech, Beijing). A NanoDrop 2000 Spectrophotometer (Thermo Scientific, United States) and a 2100 Bioanalyzer (Agilent Technologies, United States) were applied to check the RNA molecule quality, and the absorbance at 260 nm/280 nm was 1.8, and the RIN value was 9.1. cDNA was prepared using the SMARTer PCR cDNA Synthesis Kit (Clontech) from 2 μg of purified RNA. The RNA libraries were sequenced on the Illumina HiSeqTM 2000 sequencing platform.

### Expression Quantification and Differential Expression Analysis

Reads originating from RNA-seq were aligned to the reference genome using HISAT2. Fragments per kilobase of transcript per million fragments mapped (FPKM) was adopted to quantify the abundance of assembled transcripts using Stringtie and Ballgown (Pertea et al., 2015, 2016). EdgeR was applied to analyze differentially expressed genes (DEGs) in which the criteria were a twofold change (log_2_FC > 1 or < -1) in expression level and false discovery rate (FDR) < 0.05 (Nikolayeva and Robinson, 2014). The enrichment analysis of GO terms and KEGG pathways was performed using the online OmicShare tools^3^. All expressed genes were used as the background. We finally generated a total of 309,962,820 high-quality clean reads. The total mapping ratio of each sample to the genome assembly ranged from 92.78 to 93.86%, and the number of transcribed genes in each sample was predicted to range from 110,625,09 to 112,127,27 (Supplementary Table S2). FPKM values of all transcripts are provided in the Supplementary Table S3.

### Weighted Gene Coexpression Network Analysis (WGCNA)

Coexpression analysis was conducted using weighted correlation network analysis (WGCNA) (Langfelder and Horvath, 2008). After selecting 2257 DEGs between control and treatment, we log-transformed these FPKM values using log2 (FPKM value + 1) as recommended on the WGCNA FAQ’s page. We chose a soft power value β (β = 20) to approximate a scale-free network topology to generate a network, guided by a convenient 1-step network construction and module detection function in the R Tutorial^1^. Then, the Module Eigengene (ME) was calculated, which represents the expression profile of each module. Next, based on the correlation between the ME and trait, we estimated the module-trait relationships to identify highly correlated modules. The module is considered to be associated with traits, where the module-trait relationship value is ≥ 0.4 and *P* ≤ 0.05. As a basis for identifying hub genes, intramodular connectivity that was founded on the correlation between the ME and the expression profile was detected.

### qRT-PCR Validation

Total RNA from the three replicates was extracted using a TransZol Up Plus RNA Kit (Transgen Biotech, Beijing). Primers were designed using Primer Premier 5.0, and the sequences are listed in the Supplementary Table S4. qRT-PCR was performed using the Applied Biosystems 7300 real-time PCR System (Framingham, MA, United States) with SYBR Green PCR Master Mix (TaKaRa) following the procedures described previously (Borecka-Melkusova et al., 2009; Kong et al., 2014). All PCRs were performed in triplicate. 18S rRNA was used as the reference gene (Noda et al., 2004). The relative expression level was quantified by the 2^–Δ^ ^Δ^ ^Ct^ method.

## Results

### Genome Assembly and Annotation

**FIGURE 2:**
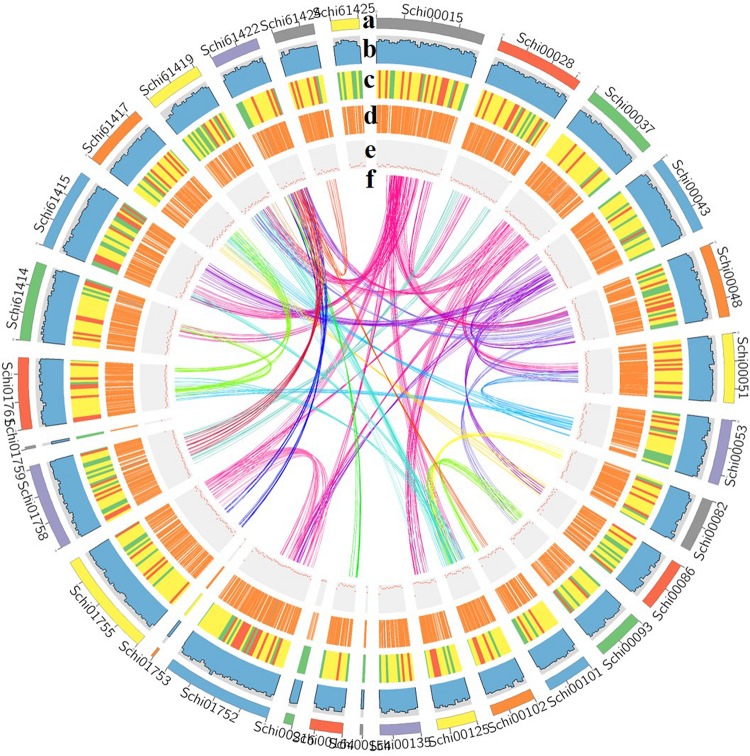
Characterization of the genome [**(a)** assembled contigs; **(b)** GC content; **(c)** gene density; **(d)** expression values of root- and shoot-expressed genes; **(e)** percent coverage of TEs in non-overlapping windows; **(f)** relationship between syntenic blocks].

A total of 6.7 Gb Pacbio subreads (∼106 × coverage of the estimated genome size) and 9.4 Gb Illumina paired-end reads (∼150 × coverage of the estimated genome size), resulting in approximately 125-fold coverage of the *S. limacinum* SR21 genome. All reads from the *S. limacinum* SR21 genome were assembled into 63 Mb, consisting of 52 contigs. The contig N50 is 2.67 Mb, and the longest contig 4,02 Mb (Figure 2 and Table 1) based on the TGS of the PacBio SEQUEL platform. The GC ratio of sequence reads was 49.9%. We further utilized the Benchmarking Universal Single-Copy Orthologs (BUSCO) software to examine the completeness of our present assembly. The results indicated that the assembled genome is of good quality (89.1% completeness) (Supplementary Table S5).

**TABLE 1:** *Schizochytrium limacinum* SR21 genome statistics.

**Estimated genome size**	**63 Mb**
G + C (%)	49.99%
Number of assembled contigs	52
Number of contigs > 2 kb	52
Contig N50 length	2.67 Mb
N rate (%)	0

A combination of reference plant protein homology support, transcriptome data, and *ab initio* gene prediction were used to generate all gene models. All gene models were merged, and redundancy was removed by MAKER, leading to a total of 6,838 protein-coding genes, with an average length of 2.4 kb. The NCBI non-redundant protein (nr) database with an e-value threshold of 1e-5 was used for functional annotation for protein-coding genes using BLASTX and BLASTN. The Blast2GO package was applied to assign ontology and pathway information to protein-coding genes using Gene Ontology (GO) and Kyoto Encyclopedia of Genes and Genomes (KEGG). Finally, we functionally annotated 6502, 5822, 3692, 5822, and 4341 genes to nr, EggNOG, GO, COG, and KEGG, respectively, leading to 6508 (95.17% of the total) genes with at least one hit in a public databases (Table 2). In total, 482 transcription factors were identified in the *S. limacinum* SR21 genome, and these genes were classified into 19 families, including 316 protein kinase family proteins, 50 MYB and 42 WD-40 repeat family proteins (Supplementary Table S6).

**TABLE 2:** Overview of genome annotation.

**Annotation statistics for genome**	**Number**	**Percent (%)**
Total protein	6838	
NR	6502	95.08
eggnog	5822	85.14
GO	3692	53.99
COG	5822	85.14
KEGG	4341	63.48
In all databases	2996	43.81
At least in one database	6508	95.17

The complete FAS genes in map00062 and map01212 were annotated, and the function of these genes enables the synthesis of fatty acids and the accumulation of DHA in *S. limacinum* SR21, including 3-ketoacyl-CoA synthase (KCS), 3-oxoacyl-CoA reductase (KAR), 3-hydroxyacyl-CoA dehydratase (HS1), enoyl-CoA reductase (ER), acyl-coenzyme A thioesterase (ACOT), 3-hydroxyacyl-CoA dehydrogenase (HADH), acetyl-CoA acyltransferase (ACAA), enoyl-CoA hydratase (ECHS), acyl-CoA dehydrogenase (ACDH),Δ-4 desaturase, Δ-5 desaturase, Δ-1 elongase, Δ-3 elongase, Δ-4 elongase, and Δ-6 elongase. Furthermore, we identified a gene cluster and 10 ORFs related to PKS pathway containing domains with homology to those in *Shewanella pneumatophori* (GenBank accession number U73935.1.), *Schizochytrium* sp. ATCC_20888 (GenBank accession number AF378327, AF378328, AF378329) and *Moritella* (GenBank accession number AB025342.1). The gene cluster (4,475 amino acids) of *S. limacinum* SR21 included typical PKS related domain, which contains the following domains:3-ketoacyl synthase (KS), malonyl-CoA acyltransferase (MAT), acyl carrier proteins (ACP), 3-ketoacyl-ACP reductase (KR), and dehydrase (DH) (Table 3).

**TABLE 3:** Summary of sequence analysis data of *Schizochytrium limacinum* SR21 genes encoding enzymes of FAS and PKS. a.a., amino acid.

**Putative function**	**Gene_id**	**Approximately size (a.a)**	**Location**	**Related to**
3-Ketoacyl synthase (KS)	schi20060510	405,919	357–762,2046–2965	PKS
	schi20014840	426	14–440	PKS
Malonyl-CoA acyltransferase (MAT)	schi20060510	206	3362–3568	PKS
	schi20008650	328	59–387	PKS
Acyl carrier proteins (ACP); phosphopantetheine attachment site (PP)	schi20060510	169,542	158–327,1469–2011	PKS
3-Ketoacyl-ACP reductase (KR)	schi20060510	265,330	1125–1390,3701–4031	PKS
Acyl transferase (AT)	schi20065660	324	28–352	PKS
	schi20039990	228	177–405	PKS
Enoyl reductase (ER)	schi20056480	321	6–327	FAS
	schi20057870	308	40–348	FAS
	schi20058990	290	33–323	FAS
	schi20000150	300	41–341	FAS
	schi20034500	283	25–308	FAS
	schi20052840	292	52–344	FAS
	schi20066510	230	89–319	FAS
Dehydrase (DH)	schi20060510	234	809–1043	FAS

### Annotation of Non-coding RNA (ncRNA)

We identified rRNA, tRNA, and snRNA genes in the *S. limacinum* SR21 genome by searching the Rfam database using BlastN with an E-value ≤ 1e-5 and predicted tRNAs and rRNAs by tRNAscan-SE and RNAmmer, resulting in an *S. limacinum* SR21 genome with 536 tRNAs, 339 rRNAs, 1 sRNA, 1 lncRNA, 1 ribozyme, and 9 snRNAs (Supplementary Table S7).

### Repeat Element (TE) Annotation

Repetitive sequences of the *S. limacinum* SR21 genome accounted for 12.47% of the assembled genome. Long terminal repeat (LTR) retrotransposons accounted for 1.83% of the genome, including 0.30% Ty1/copia, 0.10% Ty3/gypsy, and 1.44% other. The tandem repeats finder identified over 44,073 tandem repeats, accounting for 4.86% of the *S. limacinum* SR21 genome (Supplementary Table S8).

### Evolution of the *S. limacinum SR21* Genome and Gene Family Analysis

**FIGURE 3:**
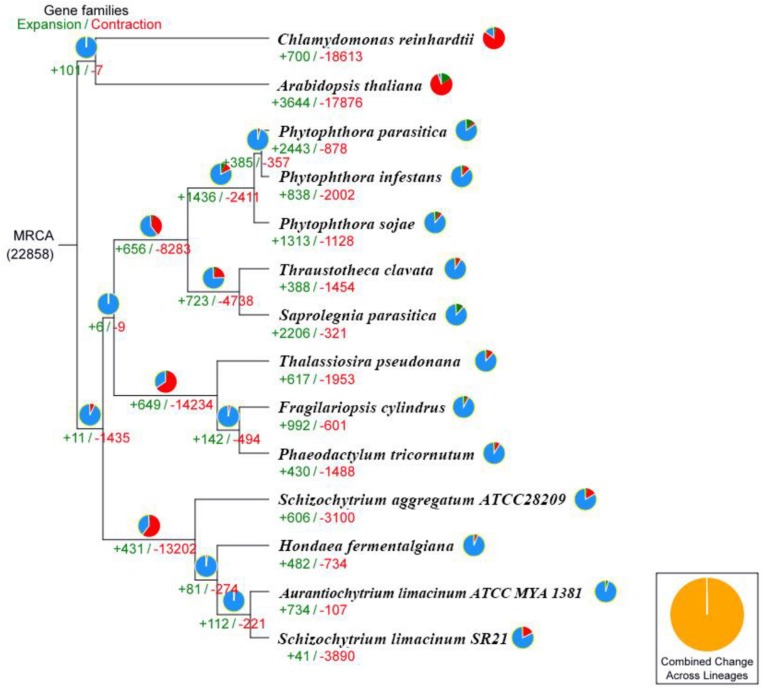
Phylogenetic tree and number of gene families displaying expansion (green) and contraction (red) among 14 plant species. The pie charts show the expanded (green), contracted (red), and conserved (blue) gene family proportions among all gene families. MRCA, most recent common ancestor.

We performed comparative genomic analyses among 14 species and detected 22,858 families of homologous genes, and among them, 1,107 gene families were common. Furthermore, 186 of the 1,107 common gene families contained one copy in each plant species. These 186 single-copy orthologous genes were used to construct the phylogenetic tree (Figure 3). The results confirmed that the strains *S. limacinum* SR21, *S. aggregatum*-ATCC28209, *A. limacinum* ATCC-MYA-1381 and *H. fermentalgiana* clustered together on the phylogenetic tree. A total of 3,931 gene families were identified in the *S. limacinum* SR21, among which 41 and 3,890 gene families showed expansion and contraction, respectively. A total of 3,890 genes in the contracted families were annotated to KEGG pathways (Supplementary Table S9) and GO terms (Supplementary Table S10), respectively. KEGG analysis found that most of the contracted gene families were clustered in signal transduction, lipid metabolism, environmental adaptation, metabolism of terpenoids and polyketides. GO analysis showed that the expanded orthogroups were related to biological regulation, catalytic activity, developmental process, metabolic process, stimulus response, signaling, and reproductive process.

**FIGURE 4:**
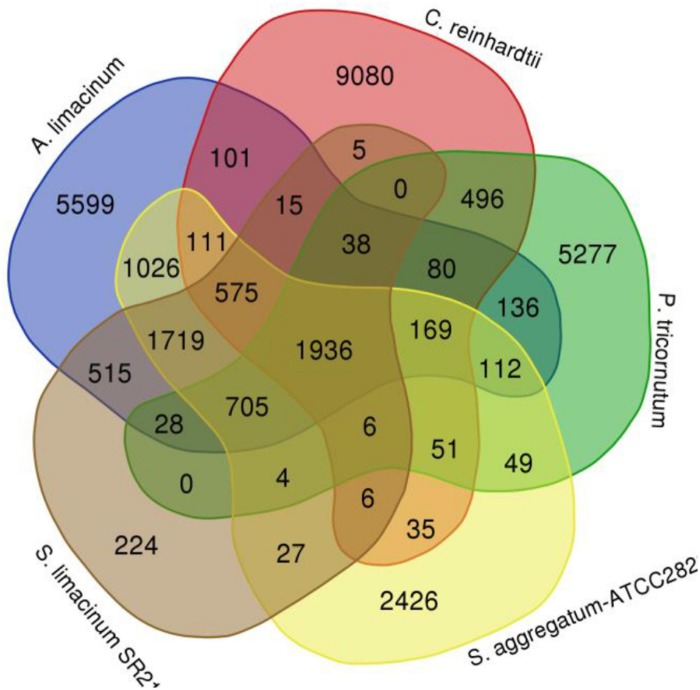
Venn diagram of gene families in *Schizochytrium limacinum* SR21 and four other species.

Compared with *S. limacinum* SR21, *A. limacinum*, *C. reinhardtii*, *P. tricornutum*, and *S. aggregatum* -ATCC28209, 1936 (33.36%) of 5,803 *S. limacinum* SR21 gene families were shared by the five species, whereas 224 gene families were unique to *S. limacinum* SR21 (Figure 4). The 244 unique families are novel gene families in *S. limacinum* SR21 during long history of evolution. Some of them may be lost in other species, while we believe that there are gene families *de novo* originated in *Schizochytrium* sp. GO enrichment analysis of these 224 unique families showed enrichment of immune system processes, biological regulation, metabolic processes, developmental processes and reproductive processes (Supplementary Table S11). KEGG analysis showed enrichment of fatty acid biosynthesis, fatty acid degradation, nitrogen metabolism and metabolic pathways (Supplementary Table S12).

**FIGURE 5:**
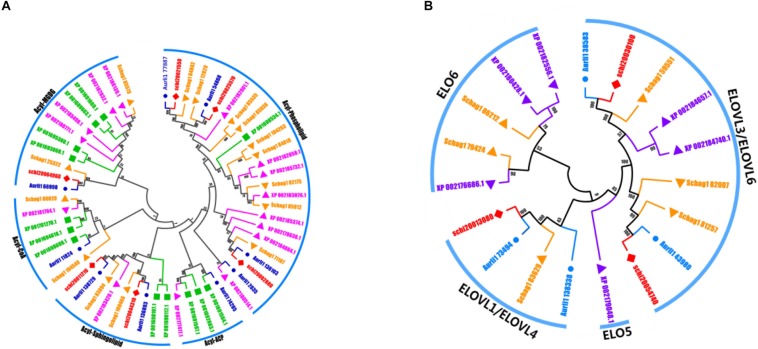
**(A)** The evolutionary tree and expression values of the desaturation gene family. **(B)** The evolutionary tree and expression values of the elongation gene family. *A. limacinum* (Auril), *S. limacinum* SR21 (schi), *P. tricornutum* (XP), and *S. aggregatum* (Schag).

Regarding LC-PUFA synthesis, it is mostly thought that polyunsaturated fatty acids in *Schizochytrium* sp. are synthesized by the FAS and PKS pathway. The desaturation-elongation proteins constitute one of the largest families of transcription factors and are involved in the regulation of the desaturation and elongation of polyunsaturated fatty acids in the FAS pathway. We identified 6 elongase and 5 desaturase subfamilies in these five species, including Δ-4 desaturase, Δ-5 desaturase, Δ-1 elongase, Δ-3 elongase, Δ-4 elongase, Δ-6 elongase, acyl-ACP, acyl-CoA, acyl-MGDG, and acyl-phospholipid. The numbers of the desaturation-elongation gene family of *S. limacinum* SR21 were much less than those of other species (Figures 5A,B).

### Transcriptome Profiling and Differentially Expressed Genes

**FIGURE 6:**
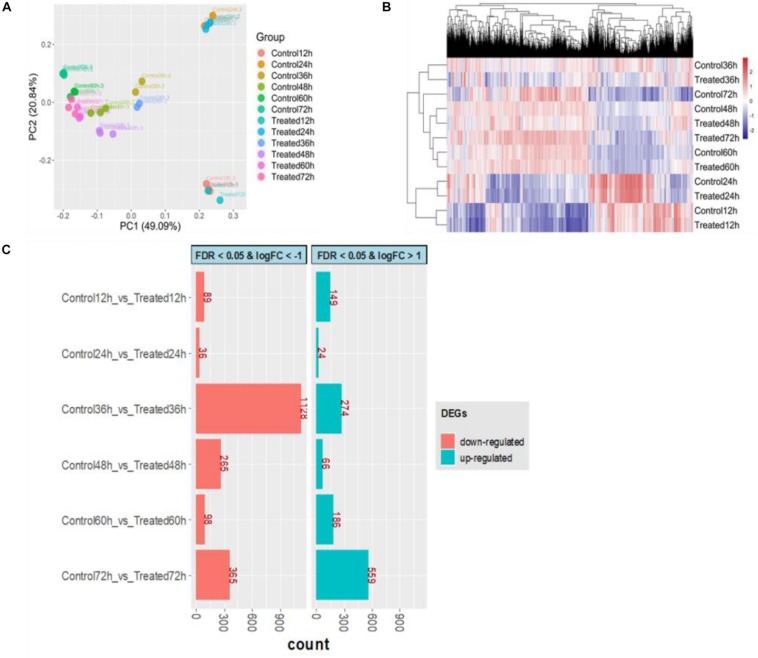
Overview of transcriptional analysis between control and treatment. **(A)** Principal component analysis for all samples. **(B)** Hierarchical clustering for transcriptome profile. **(C)** The number of differentially expressed genes (DEGs); red represents downregulated, and green represents upregulated. The control groups (Control12h, Control24h, Control36h, Control48h, Control60h, Control72h) represent that *S. limacinum* SR21 was cultured in basal fermentation medium with 1.0 g/L yeast extract at 12, 24, 36, 48, 60, and 72 h, respectively. The treated groups (Treated12h, Treated24h, Treated36h, Treated48h, Treated60h, Treated72h) represent that *S. limacinum* SR21 was cultured in basal fermentation medium with 0.5 g/L yeast extract at 12, 24, 36, 48, 60, and 72 h, respectively.

Of a total of 6,834 annotated genes, 6,632 (97.04%) genes were expressed in at least in 3 samples with a read count per million (CPM) value larger than 1. The extremely low rate (2.96%) of filtered out genes indicated that gene expression was efficiently detected across all time points. To obtain an overview of the transcriptome profile, principal component analysis (PCA) was performed by normalized log_10_(FPKM + 1) values. The first principal component (PC1), which explains 49.09% of the total variance, shows clearly different gene expression profiles between the first two early stages (12 and 24 h) and other stages (Figure 6A). The samples from the first two time points were assembled into a cluster that was distinguished from other samples, and some genes were expressed at the highest level at 24 h via the heatmap of gene expression (Figure 6B). Furthermore, to obtain insight into the DEGs involved in DHA biosynthesis and specific responsiveness to the treatment, DEGs of six pairwise comparisons between control and treatment at the same time point (control 12 h vs. treated 12 h, control 24 h vs. treated 24 h, etc.) were focused on (Figure 6C). Among these comparisons, the difference between the two transcriptomes of the control and treatment was greatest at 36 h, especially with respect to downregulated expression, indicating that 36 h might be a key time point in response to DHA biosynthesis. This result was consistent with the phenotype performance of DHA and fatty acid.

By comparing with the control 36-h group, we identified 1,402 DEGs (log2 FC ≥ | 1| and FDR ≤ 0.05) in the treated 36-h group, with 274 upregulated and 1,128 downregulated (Supplementary Table S13). Forty-two transcription factors were identified in these DEGs and were classified into 17 families, including 14 MYB, 5 WRKY, 2 C3H, and 2 C2H2 (Supplementary Table S14). GO term enrichment analysis results varied from GO classification and expression changes in DEGs. In biological processes, the upregulated DEGs were significantly enriched in biosynthetic processes, metabolic processes, stimulus responses and phosphorylation, and the downregulated DEGs were significantly enriched in protein modification processes, signal transduction and protein metabolic processes (Supplementary Table S15). KEGG pathway analysis found that 76 upregulated DEGs and 16 downregulated DEGs were enriched in the biosynthesis of secondary metabolites (Supplementary Table S16).

### Gene Coexpression Network Involved in Fatty Acid and DHA Accumulation

**FIGURE 7:**
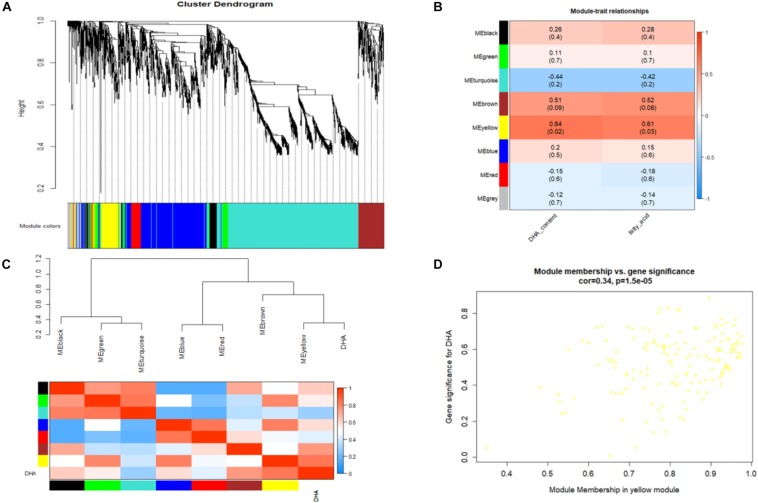
**(A)** Cluster dendrogram and module assignment from WGCNA. Each color represents a certain gene module. **(B)** Heatmap of the correlation of WGCNA modules with traits. Red is the correlation between module and trait, and blue is a negative correlation. The module highlighted with dark color represents a significant module associated with traits. **(C)** The eigengene dendrogram and heatmap for modules with DHA. **(D)** Correlation between significant genes and module membership in the yellow module.

The gene coexpression network constructed by WGCNA provides a systems biology approach to understand the gene networks instead of individual genes. Thus, WGCNA was adopted in this study to identify modules representing functional categories. According to the scale-free topology model fit and mean connectivity, a soft threshold power β was set to 20 in our study, which produced an approximate scale-free network with appropriate mean connectivity. After screening, a total of 36 samples and 2257 DEGs were used for coexpression network construction, and 7 modules with module sizes named black (75 genes), blue (535 genes), brown (194 genes), green (104 genes), red (80 genes), turquoise (1,020 genes), and yellow (155 genes) were generated (Figure 7A). Ninety-four genes were outside of those nine modules and are labeled as the gray module. Notably, analysis of the module-trait relationships revealed that the yellow and brown modules were identified as significantly highly expressed and were relevant and consistent with the results of phenotype performance of DHA and fatty acids, especially the yellow module (Figure 7B). The yellow module, DHA and fatty acid were clustered together and were highly positively related (Figure 7C). As shown in the Supplementary Table S17, a total of 9 TFs were annotated in the yellow module, and several TFs highly positively correlated with fatty and DHA accumulation were found among these 6 very-long-chain fatty acid biosynthesis regulatory genes, including three protein kinase family proteins (schi20066080, schi20050050, and schi20028430), 2 MYB (schi20061500 and schi20062190) and 1 Zinc Finger (schi20050340). According to GO enrichment analysis, unigenes in the content-related yellow module were enriched in different metabolic pathways, including lipid oxidation, lipid catabolic processes, fatty acid metabolic processes and lipid biosynthetic processes (Supplementary Table S18). KEGG enrichment analysis in the yellow module was performed to identify the key genes and pathways closely related to fatty acid and DHA accumulation (Supplementary Table S19). As we can see in the Supplementary Table S20, DEGs in the yellow module were significantly enriched in the biosynthesis of secondary metabolites, pyruvate metabolism, fatty acid degradation, and alpha-linolenic acid metabolism.

### Analysis of Hub Genes and qRT-PCR Validation

Figure 7D shows that the intramodular connectivity and module membership have a high positive linear correlation in the yellow module. In general, a high module membership corresponds to a high intramodular connectivity. Genes with both of these properties may be important candidate hub genes. In order to identify the hub genes that well-represent the yellow module, we analyzed the module in further detail by GO and KEGG pathway (Supplementary Figure S1). The yellow module has 155 total genes, and the top 30 genes are listed in the Supplementary Table S20 and Supplementary Figure S2. Hub gene analysis identified acyl-CoA oxidase (schi20049930) and *N*-ethylmaleimide reductase (schi20023280) in the yellow module, which are involved in fatty acid beta-oxidation, very-long-chain fatty acid metabolic processes and oxidation-reduction processes. In order to confirm the accuracy of unigene expression levels, three unigenes from the yellow module and RNA-Seq data were selected for qPCR analysis, and their relative expression levels were compared with FPKM values from RNA-Seq data. The results showed that the expression of all three unigenes measured by qPCR was consistent with the RNA-Seq data (Supplementary Figure S3).

## Discussion

Four DHA-producing thraustochytrid strains (*Schizochytrium* sp. CCTCC M209059, *Aurantiochytrium* sp. T66, *Schizochytrium* sp. Mn4, and *Thraustochytriidae* sp. SW8) have been produced based on next-generation sequencing platforms or a small number of PacBio RS (Ji et al., 2015; Liu et al., 2016; Song et al., 2018). However, short sequencing reads typically cannot span highly repetitive segments of genomes and be assembled into sets of small contigs (scaffold N50 127 kb to 1.3 Mb). In order to produce a high-quality genome, we generated a draft genome assembly with 63 Mb in total length and 52 contigs (>2,000 bp) with a high contig N50 of 2.67 Mb. A large number of protein-coding genes (6,838) was predicted by the gene models built with *de novo*, homology-based, and experimental data obtained from transcription results. These findings indicated that a high-quality *S. limacinum* SR21 genome was generated, which provided a valuable reference for understanding the molecular synthesis and regulatory networks for PUFA biosynthesis in *Schizochytrium* sp.

Based on the concatenated sequence alignment of *S. limacinum* SR21 and 13 other species, the strains *S. limacinum* SR21, *S. aggregatum*-ATCC28209, *A. limacinum* ATCC-MYA-1381 and *H. fermentalgiana* clustered together on the phylogenetic tree. A total of 3,931 gene families were identified in the *S. limacinum* SR21, and the number of contracted gene families (3,890) is significantly greater than that of expanded gene families (41). KEGG and GO analyses showed that the gene families involved glycerophospholipid metabolism, glycerolipid metabolism, cutin, suberine, and wax biosynthesis have significantly contracted in the *S. limacinum* SR21 genome, which is not directly related to fatty acid biosynthesis.

LC-PUFAs can be synthesized by fatty acid synthases pathway (FAS) and polyketide synthases pathway (PKS). Currently, the two major steps in fatty acid biosynthesis were clarified as elongation and desaturation carried out by an elongase and desaturase in the FAS pathway (Morais et al., 2015). Lippmeier et al. (2009) found Δ-5, Δ-6 and Δ-9 elongase activities in *Schizochytrium* sp. ATCC20888 without the present of Δ-12 desaturation. Ren et al. (2017), detected one elongase and three kinds (Δ-6, Δ-8, and Δ-12) of desaturase activities in *Schizochytrium* sp. The missing of some specific enzymes might be the reason of the incomplete genomic information. In our research, we identified two unigenes encoding desaturase (Δ-4 desaturase, Δ-5 desaturase) and four unigenes encoding elongase protein (Δ-1 elongase, Δ-3 elongase, Δ-4 elongase, Δ-6 elongase), and the all genes needed for FAS pathway in map00062 and map01212 were annotated (Supplementary Figure S4). Similar to Lippmeier’s results, we also find Δ-6 elongase which catalyzes C18:4 to C20:4, indicating *Schizochytrium* was able to convert SDA to ARA. Different from Lippmeier’s and Ren’s results, we annotated Δ-4 desaturase which coverts C22:5 to C22:6, indicating *Schizochytrium* was able to catalyze DPA to DHA and played important role in the fatty acid synthesis. Maybe it is the reason why *Schizochytrium* sp. can accumulate rich LC-PUFAs and DHA. However, *Schizochytrium* sp. is considered as the best natural resource for LC-PUFAs and more than 50% lipid rich in DHA. The questions remain as to how to accumulate rich LC-PUFAs and promote DHA production with the relatively low numbers of desaturase and elongase gene families in *S. limacinum* SR21 compared with other four species. We assumed that the gene members of these families have stronger catalytic capabilities or that there are some strong transcriptional regulators that regulate the ability of these functional genes to enhance fatty acid synthesis.

**FIGURE 8:**
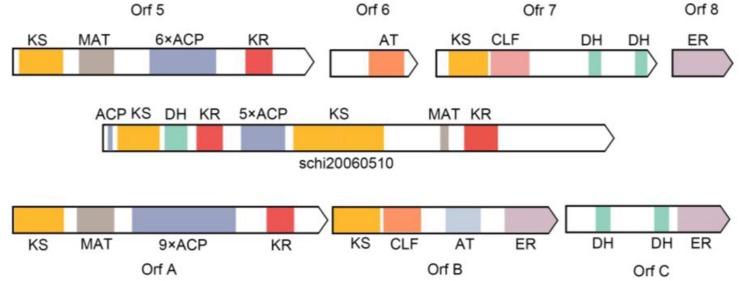
Comparison of putative PKS enzyme domains in *Shewanella pneumatophori* (GenBank accession number U73935.1.), *Schizochytrium* sp. ATCC_20888 (GenBank accession number AF378327, AF378328, AF378329) and *Schizochytrium limacinum* SR21 (schi20060510). 3-ketoacyl synthase (KS), malonyl-CoA acyltransferase (MAT), acyl carrier proteins (ACP), 3-Ketoacyl-ACP reductase (KR), enoyl reductase (ER), and dehydrase (DH).

Metz (2001), identified 11 domains from *Schizochytrium* sp. by comparing with *Shewanella* sp. domains, predicted eight gene products, closely related to the PKS protein domain (Metz, 2001). The prokaryotic *Shewanella* sp. and eukaryotic *Schizochytrium* sp. genes have high homology, and the gene structure and functional regions are similar. In PKS pathway for LC-PUFAs synthesis, the pathway catalyzed by polyketide does not require desaturation and elongation of saturated fatty acids, the synthases for PKS are totally different from PKS in both structure and mechanism. They concluded that PUFA synthesis in *Schizochytrium* sp. is accomplished in part by these PKS enzymes. Until now, although the related domains of the PKS pathway have been cloned and annotated in the comparison with the related domains of the PKS pathway of bacteria, the fatty acid biosynthesis has not been still clarified whether through the FAS or PKS pathway mainly in *Schizochytrium* sp. We identified one gene cluster and 10 ORFs related to PKS pathway containing domains with homology to those in *Shewanella pneumatophori*, *Schizochytrium* sp. ATCC_20888, and *Moritella*. The gene cluster (4,475 amino acids) of *S. limacinum* SR21 included typical PKS related domain, including 3 KS, 1 MAT, 6 ACP, 2 KR, and 1 DH (Figure 8). In addition, we also annotated some domains related to PKS and FAS in the genome of *S. limacinum* SR21, including 1 KS, 7 ER, 2 AT, and 1 MAT (Table 3). These genes are individually distributed on the genome, not in cluster. And the function of these genes needs to be figured out.

Yeast extract starvation are the premise of lipid production and DHA accumulation. In our previous study, the total fatty content and DHA production of *S. limacinum* SR21 drastically increased from 12 to 36 h in basal fermentation medium containing 1.0 or 0.5 g/L yeast extract, respectively. The R2R3-type MYB96 transcription factor is a pivotal regulator of fatty acid elongation, which regulates cuticular wax accumulation under drought conditions in *Arabidopsis* leaves (Seo et al., 2011). Lee H. G. et al. (2015), reported that MYB96 could directly regulate fatty acid elongation to stimulate accumulation of eicosenoic acid in *Arabidopsis* seed maturation and development. In our study, most of DEGs were downregulated after nitrogen limitation at 36 h and 42 transcription factors were identified and classified into 17 families in these DEGs, including 14 MYB, 5 WRKY, 2 C3H, and 2 C2H2. A weighted gene coexpression network analysis revealed that 2 of 7 modules correlated highly with the fatty acid and DHA contents, and DEGs and transcription factors were significantly correlated with fatty acid biosynthesis, including MYB, Zinc Finger and ACOX. AtMYB12, AtMYB111, AtMYB11, and MdMYB22 have been shown to be involved in flavonoid biosynthesis and anthocyanin biosynthesis in *Arabidopsis*, tobacco and apple (Park et al., 2008; Lee K. et al., 2015; Tian et al., 2017). These studies suggest that the identified TFs may be involved in nitrogen limitation-induced accumulation and regulation of LC-PUFAs and DHA production in *S. limacinum* SR21, likely working together with a transcription factor complex. Interestingly, several studies have suggested a close relationship between fatty acid and TAG synthesis. ACOX (acyl-CoA oxidase) catalyzes the first step in the pathway of peroxisomal fatty acid beta-oxidation, which is part of lipid metabolism, catalyzing the desaturation of acyl-CoAs to 2-*trans*-enoyl-CoAs (Kim and Kim, 2018). Here, the WGCNA distributed the ACOX1 gene from the fatty acid biosynthetic pathway into the yellow module. Furthermore, qRT-PCR confirmed that ACOX1 was strongly induced by nitrogen limitation treatment. We also found that these DEGs and hub genes are involved in the encoding enzymes of PKS pathway based on the results of transcriptome and weighted gene coexpression network analysis. Therefore, we hypothesize that nitrogen limitation can accumulate DHA and fatty acid contents by activating the fatty acid beta-oxidation process in FAS pathway. The results can provide a basis for distinguishing which pathways of fatty acid biosynthesis is mainly achieved through, and we can regulate FAS pathways to increase the production of fatty acids and DHA in *Schizochytrium* sp. The results should help improve the accumulation of fatty acid and DHA in *Schizochytrium* sp. and other microalgae, providing a valuable reference for industrial applications.

## Data Availability Statement

The whole genome sequence data reported in this article have been deposited in the Genome Warehose in National Genomics Data Center, Beijing Institute of Genomics (BIG), Chinese Academy of Sciences, under accession number GWHABLD00000000 that is publicly accessible at https://bigd.big.ac.cn/gwh. Raw sequencing data for RNA-seq was used for annotation and biological analyses, and have also been deposited in BIG Sub system under BioProject accession number PRJCA002397 (http://bigd.big.ac.cn).

## Author Contributions

YC and TX designed and coordinated the entire project. TX, YC, WT, and JZ together led and performed the entire project. LL, XZ, and ZH performed the collection and processing of samples. TX, XZ, WF, DC, JP, and WT performed the analyses of genome evolution and gene family analyses. TX, LL, XZ, DC, and YC participated in manuscript writing and revision. All authors read and approved the final manuscript.

## Conflict of Interest

The authors declare that the research was conducted in the absence of any commercial or financial relationships that could be construed as a potential conflict of interest. The reviewer FQ declared a shared affiliation, with no collaboration, with the authors to the handling editor at the time of review.

## Footnotes

1 https://horvath.genetics.ucla.edu/html/CoexpressionNetwork/Rpackages/WGCNA/Tutorials/

2 http://www.omicshare.com/tools

3 http://www.omicshare.com/tools
