# Supplementary material for: Genome and Transcriptome Analyses Provide Insight Into the Omega-3 Long-Chain Polyunsaturated Fatty Acids Biosynthesis of Schizochytrium limacinum SR21
Source: Front Microbiol. 2020 Apr 16;11:687. doi: 10.3389/fmicb.2020.00687 (PMC7179369; doi:10.3389/fmicb.2020.00687)
Supplement: Supplementary file 1 [file Data_Sheet_1.doc]

**Supporting Information Tables**

Table S1. Sequencing data used for the *S. limacinum* SR21 genome construction. Note that the sequence coverage were calculated using the Kmer based estimated genome size.

| Library  resource | Sequencing platform | Insert size (bp) | Clean data (Gb) | Sequence coverage (X) | Usage of the data |
| --- | --- | --- | --- | --- | --- |
| genome | Illumina HiSeq X Ten | 250 bp | 9.4 | 150 | Genome estimation and polish |
| genome | PacBio SEQUEL | 20 kb | 6.7 | 106 | Genome assembly |
| transcriptome | PacBio SEQUEL | 0.6-3 kb | 44.3 | - | Difference analysis and annotation |

Table S2. Mapping data of each transcriptome sample to the generated genome assembly.

| Samples | Total reads | Overall  alignment rate | Unmapped reads | Unique mapped reads |
| --- | --- | --- | --- | --- |
| Control-12h | 10088436 | 96.73% | 5.57% | 93.36% |
| Control-24h | 11493948 | 97.28% | 5.07% | 92.98% |
| Control-36h | 11196209 | 97.02% | 4.83% | 93.34% |
| Control-48h | 11431437 | 96.94% | 5.21% | 92.93% |
| Control-60h | 11212727 | 97.36% | 4.69% | 93.86% |
| Control-72h | 11143208 | 97.06% | 5.02% | 93.29% |
| Treated-12h | 11063315 | 97.01% | 5.35% | 93.41% |
| Treated-24h | 11062509 | 96.97% | 5.07% | 92.78% |
| Treated-36h | 10416372 | 97.14% | 4.96% | 93.42% |
| Treated-48h | 11340903 | 97.22% | 4.83% | 93.61% |
| Treated-60h | 11273594 | 96.44% | 5.55% | 93.21% |
| Treated-72h | 11455652 | 97.29% | 4.74% | 93.52% |

Table S3. Gene transcription levels (FPKM values) in the each sample.

|  | FPKM=0 | 0<FPKM<1 | 1<=FPKM<5 | 5<=FPKM<10 | 10<=FPKM<20 | 20<=FPKM<50 | 50<=FPKM |
| --- | --- | --- | --- | --- | --- | --- | --- |
| Control-12h | 172 | 356 | 1095 | 915 | 1078 | 1340 | 1677 |
| Control-24h | 57 | 117 | 691 | 866 | 1195 | 1564 | 2142 |
| Control-36h | 81 | 200 | 682 | 735 | 1186 | 1651 | 2097 |
| Control-48h | 172 | 385 | 659 | 623 | 1043 | 1615 | 2135 |
| Control-60h | 449 | 418 | 631 | 589 | 975 | 1533 | 2037 |
| Control-72h | 472 | 485 | 740 | 612 | 913 | 1364 | 2045 |
| Treated-12h | 154 | 403 | 1145 | 899 | 1042 | 1298 | 1690 |
| Treated-24h | 65 | 151 | 797 | 890 | 1131 | 1493 | 2105 |
| Treated-36h | 210 | 492 | 1099 | 836 | 1003 | 1258 | 1734 |
| Treated-48h | 246 | 467 | 686 | 632 | 1049 | 1513 | 2039 |
| Treated-60h | 174 | 370 | 861 | 786 | 1061 | 1421 | 1959 |
| Treated-72h | 210 | 443 | 882 | 751 | 1038 | 1398 | 1911 |

Table S4. Primers were designed for qRT-PCR

| Genes | Sequences |
| --- | --- |
| 18s-F | AGGCGCGTAAATTACCCACT |
| 18s-R | GTCGCCCATGCCAGAAATTC |
| OPR-F | TGAGCTATCCCACCGCATAG |
| OPR-R | GCCCTCGGTTTGATCTTTGG |
| KARI-F | CAGACACTACAAAGGCGCTG |
| KARI-R | CACCAACTTGACCCATGCTC |
| MYB-F | CTCATTCCTGGTCGCATTGG |
| MYB-R | CTTCACAGCGTTCTCAGAGC |

Table S5 Assessment of the completeness of the *S. limacinum* SR21 genome assembly by BUSCO

| Type | Number | Percent (%) |
| --- | --- | --- |
| Complete BUSCOs (C) | 270 | 89.1 |
| Complete and single-copy BUSCOs (S) | 262 | 86.5 |
| Complete and duplicated BUSCOs (D) | 8 | 2.6 |
| Fragmented BUSCOs (F) | 3 | 1.0 |
| Missing BUSCOs (M) | 30 | 9.9 |
| Total BUSCO groups searched | 303 | 100 |

Table S6 Transcription factors

| Number | Classify |
| --- | --- |
| 316 | protein kinase family protein |
| 50 | MYB family protein |
| 42 | WD-40 repeat family protein / zfwd4 protein (ZFWD4) |
| 20 | DNAJ heat shock N-terminal domain-containing protein |
| 14 | C2H2-type zinc finger family protein |
| 7 | CCCH-type zinc finger protein with ARM repeat domain |
| 6 | nucleic acid binding;zinc ion binding;DNA binding |
| 4 | ethylene induced calmodulin binding protein |
| 4 | nuclear factor Y |
| 3 | Calmodulin-binding transcription activator protein with CG-1 and Ankyrin domains |
| 3 | response regulator |
| 2 | APRATAXIN-like |
| 2 | Tesmin/TSO1-like CXC domain-containing protein |
| 2 | abscisic acid responsive elements-binding factor 2 |
| 2 | calmodulin binding;transcription regulators |
| 2 | heat shock transcription factor |
| 1 | basic helix-loop-helix (bHLH) DNA-binding superfamily protein |
| 1 | basic leucine-zipper 44 |
| 1 | pentatricopeptide (PPR) repeat-containing protein |

Table S7 Statistical analysis of non-coding RNAs in *S. limacinum* SR21 genome

| Class | number | totalLen | meanLen |
| --- | --- | --- | --- |
| rRNAs | 339 | 535001 | 1578 |
| tRNAs | 536 | 41663 | 77 |
| sRNAs | 1 | 296 | 296 |
| snRNAs | 9 | 1468 | 72 |
| lncRNA | 1 | 155 | 155 |
| ribozyme | 1 | 363 | 363 |

Table S8 Repetitive element annotations in the *S. limacinum* SR21

|  | No. of TEs | Length (bp) | % of genome |
| --- | --- | --- | --- |
| Total repeat fraction | 53693 | 7854964 | 12.47 |
| Class I: Retroelement | 4592 | 1967387 | 3.12 |
| LTR Retrotransposon | 1930 | 1153190 | 1.83 |
| Ty1/Copia | 167 | 189309 | 0.30 |
| Ty3/gypsy | 39 | 58377 | 0.09 |
| Other | 1724 | 905504 | 1.44 |
| Non-LTR Retrotransposon | 1890 | 567601 | 0.90 |
| LINE | 1364 | 428926 | 0.68 |
| SINE | 526 | 138675 | 0.22 |
| Class II: DNA Transposon | 1021 | 925784 | 1.47 |
| PIF/Harbinger | 19 | 16604 | 0.03 |
| Other | 1002 | 909180 | 1.44 |
| Tandem Repeats | 44073 | 3063498 | 4.86 |

Table S17 Transcription factors

| Number | Classify |
| --- | --- |
| 3 | protein kinase family protein |
| 1 | Calmodulin-binding transcription activator protein with CG-1 and Ankyrin domains |
| 1 | DNA binding;zinc ion binding;nucleic acid binding;nucleic acid binding |
| 1 | myb domain protein 3r-5 |
| 1 | myb domain protein 3r2 |
| 1 | pentatricopeptide (PPR) repeat-containing protein |
| 1 | zinc finger (C2H2 type) family protein |
