# Supplementary material for: Genome and Transcriptome Analyses Provide Insight Into the Omega-3 Long-Chain Polyunsaturated Fatty Acids Biosynthesis of Schizochytrium limacinum SR21
Source: Front Microbiol. 2020 Apr 16;11:687. doi: 10.3389/fmicb.2020.00687 (PMC7179369; doi:10.3389/fmicb.2020.00687)
Supplement: Supplementary file 3 [file Data_Sheet_3.doc]

Supporting Information Figures

A B







Supplementary figure S1 A) GO enrichment of DEGs significantly genes in the yellow module. B) KEGG enrichment of DEGs significantly genes in the yellow module.

A B







Supplementary figure S2 A) GO enrichment of top 30 DEGs significantly genes in the yellow module. B) KEGG enrichment of top 30 DEGs significantly genes in the yellow module.

A ACOX


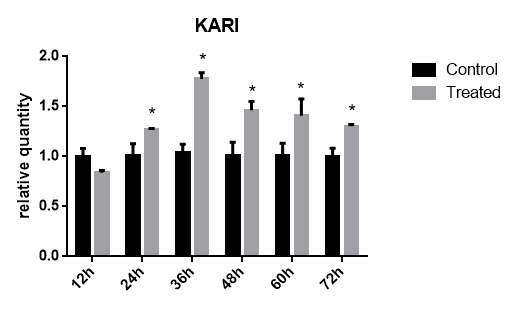


B N-ethylmaleimide reductase


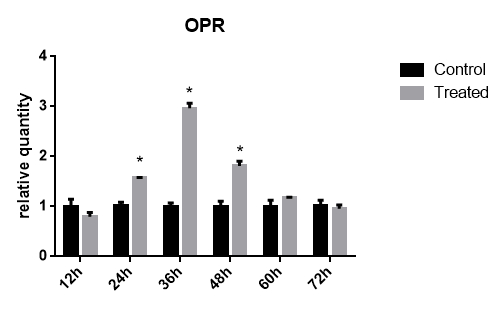


C MYB


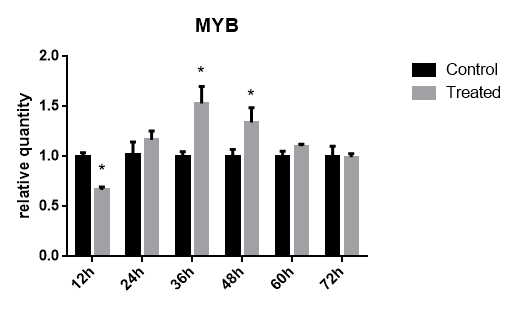


Supplementary figure S3 Expression profile of three genes involved in fatty acid biosynthesis revealed by quantitative real time PCR (qRT-PCR). Error bars indicate standard deviation. Tukey’ multiple comparison test was used when the ANOVA detected significant differences (p < 0.05) between strains.


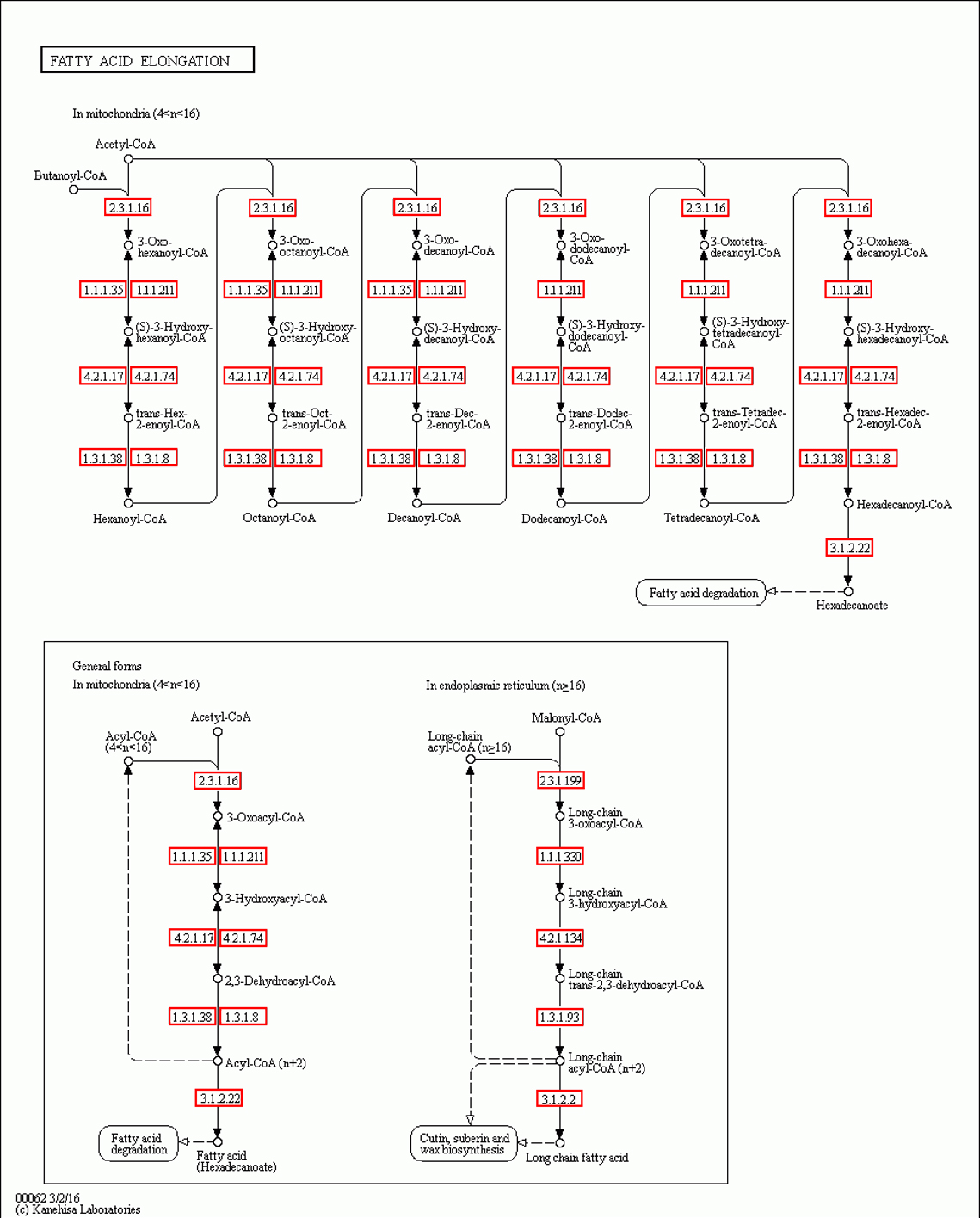


Supplementary figure S4 Related genes were annotated in FAS of *S. limacinum* SR21 (red genes: annotated)
